# Heterotrimeric G-Protein, G_α16_, Is a Critical Downstream Effector of Non-Canonical Wnt Signaling and a Potent Inhibitor of Transformed Cell Growth in Non Small Cell Lung Cancer

**DOI:** 10.1371/journal.pone.0076895

**Published:** 2013-10-18

**Authors:** Sreedevi Avasarala, Rama Kamesh Bikkavilli, Michelle Van Scoyk, Wei Zhang, Ajibike Lapite, Logan Hostetter, Joshua T. Byers, Lynn E. Heasley, Jang Won Sohn, Robert A. Winn

**Affiliations:** 1 Department of Pulmonary, Critical Care, Sleep and Allergy, College of Medicine, University of Illinois at Chicago, Chicago, Illinois, United States of America; 2 Department of Pediatrics, College of Medicine, University of Illinois at Chicago, Chicago, Illinois, United States of America; 3 Department of Craniofacial Biology, Anschutz Medical Campus, University of Colorado, Denver, Colorado, United States of America; 4 Department of Internal Medicine, School of Medicine, Hanyang University, Seoul, South Korea; 5 Jesse Brown Veterans Affairs Medical Center, Chicago, Illinois, United States of America; Virginia Commonwealth University, United States of America

## Abstract

G-protein-coupled receptors (GPCR) are the largest family of cell surface molecules that play important role/s in a number of biological and pathological processes including cancers. Earlier studies have highlighted the importance of Wnt7a signaling via its cognate receptor Frizzled9, a GPCR, in inhibition of cell proliferation, anchorage-independent growth, and reversal of transformed phenotype in non small cell lung cancer primarily through activation of the tumor suppressor, PPARγ. However, the G-protein effectors that couple to this important tumor suppressor pathway have not been identified, and are of potential therapeutic interest. In this study, by using two independent Wnt7a/Frizzled9-specific read-outs, we identify G_α16_ as a novel downstream effector of Wnt7a/Frizzled9 signaling. Interestingly, G_α16_ expression is severely down-regulated, both at the messenger RNA levels and protein levels, in many non small cell lung cancer cell lines. Additionally, through gene-specific knock-downs and expression of GTPase-deficient forms (Q212L) of G_α16_, we also establish G_α16_ as a novel regulator of non small cell lung cancer cell proliferation and anchorage-independent cell growth. Taken together, our data not only establish the importance of G_α16_ as a critical downstream effector of the non-canonical Wnt signaling pathway but also as a potential therapeutic target for the treatment of non small cell lung cancer.

## Introduction

Wnts are secreted glycoproteins, which transduce key signal transduction events that play critical roles not only during mammalian development but also in many human diseases [1]. Wnts bind to the Frizzled receptors (Fzds), and activate either a canonical or β-catenin dependent pathway or non-canonical or β-catenin independent pathways via c-Jun N-terminal kinase (JNK), p38 mitogen activated protein kinase (MAPK) pathway or peroxisome proliferator-activated receptor γ pathways (PPARγ) [2]–[7]. Aberrant activation of Wnt signaling has been implicated in many diseases including cancer [1], [8]. We have previously identified that Wnt7a is lost in non-small cell lung cancers (NSCLC) [5], [6], and restoration of Wnt7a signaling in NSCLC cell lines leads to reversal of transformed phenotype [6], unveiling Wnt7a signaling as a novel tumor suppressive pathway in lung cancer. However, the mechanism of Wnt7a signal transduction from the plasma membrane to the cytoplasm and nucleus remains largely unknown.

The superfamily of G-protein-coupled receptors (GPCRs) is the largest known family of proteins in the mammalian genome [9] and their dysfunction is associated with a number of prevalent human diseases. In fact, emerging experimental and clinical data indicate that GPCRs have a critical role not only in cancer progression and metastasis, but also in many other human diseases, making GPCRs the largest targets for current therapeutic agents [10]. It has previously been shown that GPCRs are associated with autocrine growth in Small Cell Lung Cancer (SCLC, [11], [12]). Frizzleds are rightly included in the G-protein-coupled receptor (GPCR) superfamily as they display seven transmembrane domain structure, sensitivity to pertussis toxin and modulation of intracellular calcium. Interestingly, there are ten different Fzds cloned thus far. Although, all the Fzd receptors display similar heptahelical structure, it remains elusive how these receptors signal to different downstream effectors. Another cardinal property of GPCRs is that they signal via heterotrimeric G-proteins [13]–[19], implying that heterotrimeric G-proteins might modulate the effects of Fzds.

We have previously shown that restoration of Wnt7a/Fzd9 signaling inhibited both cell proliferation and anchorage-independent growth, promoted cellular differentiation, and reversed the transformed phenotype in NSCLC cells via the activation of PPARγ and stimulation of E-cadherin proteins [5], [20]. However, the G-protein/s mediating the anti-tumorigenic role of Wnt7a/Fzd9 signaling remains unknown. In this study, we utilized Wnt7a-stimulated PPARγ and E-cadherin activation as readouts and identify the heterotrimeric G-protein, G_α16_, as an important downstream effector of Wnt7a/Fzd9 signaling. Interestingly, we also observe reduced expression of G_α16_; both at the transcript level and at the protein level, in many NSCLC cell lines. Additionally, using gene specific knock downs and expression of constitutively active mutants of G-proteins, we also demonstrate that G_α16_ is critical for Wnt7a/Fzd9–mediated inhibition of transformed growth in NSCLC. Furthermore, we also establish G_α16_ as a novel mediator of Wnt7a/Fzd9-mediated activation of ERK5 and nuclear receptor tumor suppressor PPARγ. Taken together, G_α16_ is shown here to be a novel regulator of NSCLC cell proliferation and anchorage-independent cell growth.

## Results

### Identification of Heterotrimeric G-proteins Regulating Wnt7a/Fzd9 Signaling

**Figure 1:**
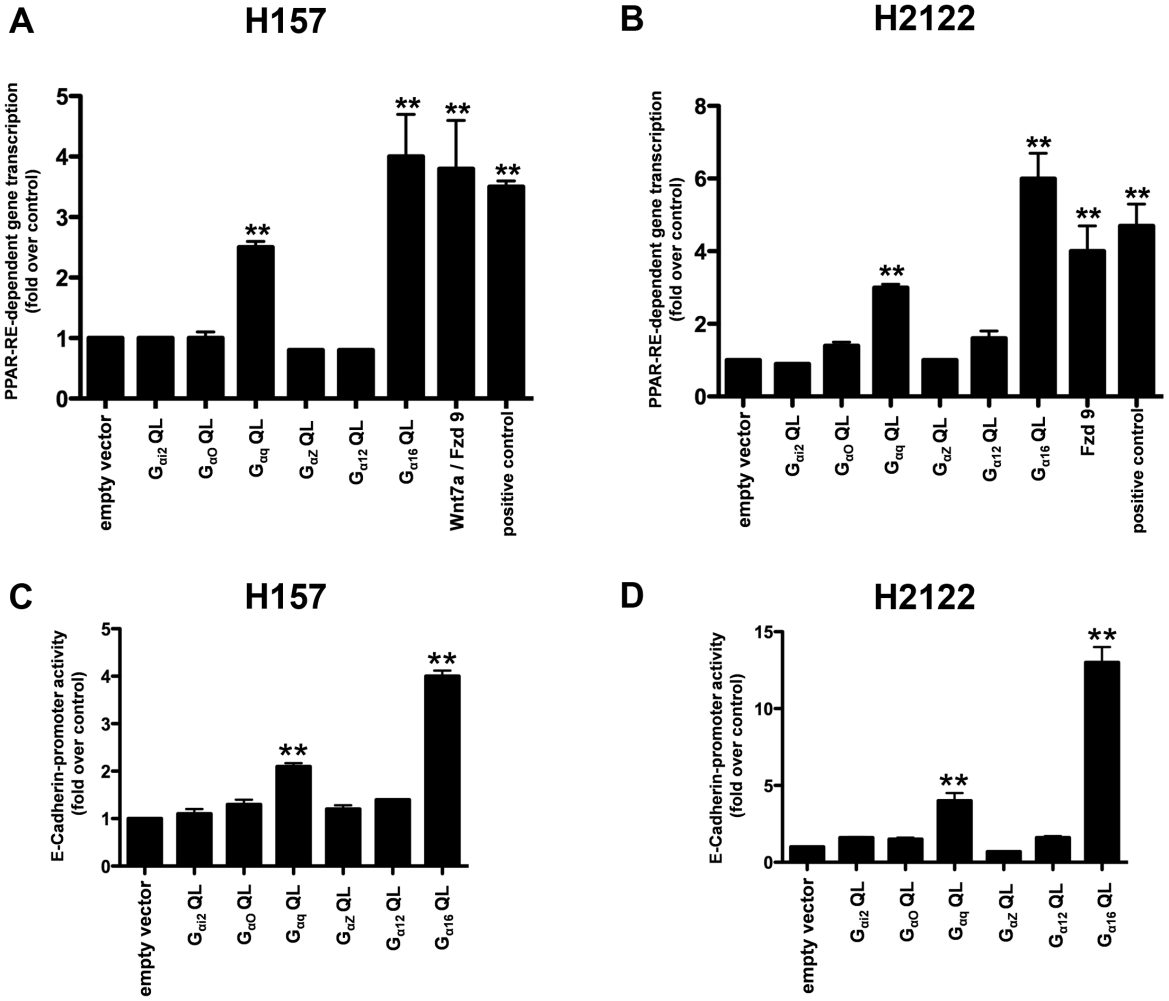
Identification of G_α16_ as a novel downstream regulator of Wnt7a/Fzd9 signaling. Effects of constitutively active G_α_ subunits on Wnt7a/Fzd9-dependent read-outs. NSCLC cell lines, H157 (A) or H2122 (B) were transfected either with empty vector, or constitutively active G_α_ subunits of G-proteins together with PPAR-RE-luciferase reporter and CMV-β-galactosidase reporter vectors. After 48 h, the cells were lysed and luciferase activities were measured as described in the Methods. NSCLC cell lines, H157 (C) or H2122 (D) were transfected either with empty vector, or constitutively active G_α_ subunits of G-proteins together with E-cadherin promoter-luciferase-reporter and CMV-β-galactosidase reporter vectors. After 48 h, the cells were lysed and luciferase activities were measured as described in the Methods. Luciferase values were normalized to CMV-β-galactosidase values and were represented in the graph. Constitutively active G_α_ subunit-induced PPRE-dependent gene transcription or E-cadherin promoter activity were represented as the fold change over the empty vector control. Data represents mean ± SEM of three separate experiments. **, *p*<0.01; versus empty vector control.

To evaluate the possible involvement of G-protein/s in Wnt7a/Fzd9 signaling, we made use of constitutively active G_α_ subunits of G-proteins that are deficient in GTPase activity, and probed their effects on two well established Wnt7a/Fzd9-dependent read-outs *viz.,* PPAR-dependent gene transcription and E-cadherin-dependent gene transcription in NSCLC cell lines [5], [20]. NSCLC cell lines (H157 and H2122) were transiently transfected with either an empty vector or a panel of constitutively active G_α_ subunits of G-proteins (G_αi2_Q205L, G_αo_Q205L, G_αq_Q209L, G_αz_Q205L, G_α12_Q229L, or G_α16_Q212L) together with a PPAR-Response Element (RE) luciferase reporter vector. The effects of the expression of constitutively active G-proteins on PPAR-dependent gene transcription were later determined by measuring luminescence in the cell lysates (Fig. 1A, B). Interestingly, expression of G_α16_Q212L, but not G_αi2_Q205L, G_αo_Q205L, G_αz_Q205L, or G_α12_Q229L, resulted in a four-fold increase in PPAR-RE luciferase activity in both the cell lines tested, an effect similar to that of Wnt7a/Fzd9-stimulated PPAR-RE luciferase activity alone (Fig. 1A, B). For the positive controls, since, H157 and H2122 cells have reduced or no Wnt7a expression, the cells were transfected with Wnt7a expression plasmids [5], [20]. H157 cells were additionally transfected with Fzd9 plasmid, as they do not to express endogenous Fzd9 [5], [20]. It was also interesting to note that expression of G_αq_Q209L also induced PPAR-RE-luciferase activity, *albeit* less efficiently than G_α16_Q212L (Fig. 1A, B). The effects of either G_α16_Q212L or G_αq_Q209L expression were specific to PPARγ activity but not to PPARδ activity, since, the expression of G_α16_Q212L, G_αq_Q209L, or Wnt7a/Fzd9 in H157 and H2122 failed to stimulate PPARδ-RE luciferase activity, a specific reporter for PPARδ (data not shown). Since, Wnt7a/Fzd9 signaling failed to stimulate PPARα activity (data not shown), we therefore did not attempt to test the effects of G_α16_Q212L, G_αq_Q209L on PPARα activation.

E-cadherin is a well-known marker of epithelial differentiation [21], [22] and has previously been shown to be an important downstream target for both Wnt7a/Fzd9 signaling and PPARγ expression in NSCLC [6], [23]. We therefore also evaluated the effects of constitutively active G-proteins on E-cadherin promoter activity in NSCLC cells (H157 and H2122). Similar to the effects on PPARγ activity, expression of G_α16_Q212L induced a robust increase in E-cadherin promoter activity in both the cell lines tested when compared to empty vector controls (Fig. 1C, D). The effects of G_αq_Q209L expression on E-cadherin promoter activity, although significant, were less potent than that of G_α16_Q212L expression (Fig. 1C, D). Taken together, these results suggest a strong association of the G-proteins, G_α16_ and G_αq_, with activation of PPARγ and increased cellular differentiation as shown by increased E-cadherin expression.

### G_α16_ Expression is Lost in NSCLC

**Figure 2:**
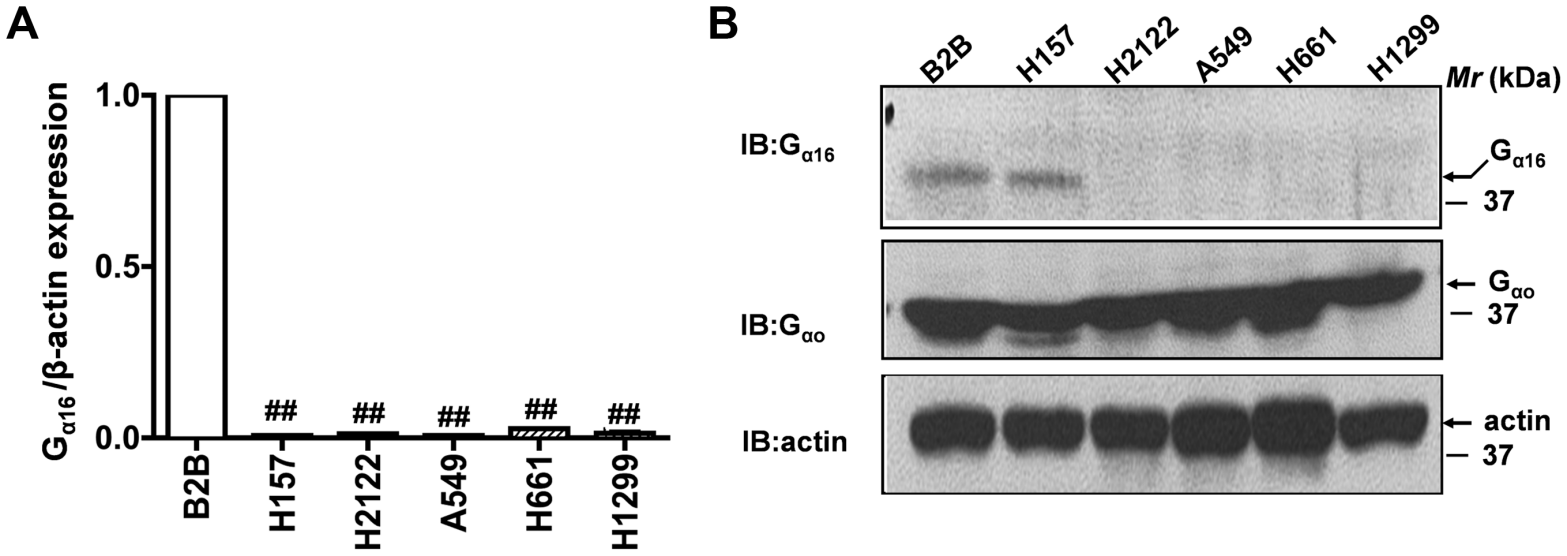
G_α16_ expression is lost in NSCLC. A, Real-time PCR analyses of the expression of G_α16_ in non-transformed and NSCLC cell lines. Total RNA was extracted from a non-transformed cell line (Beas2B) or NSCLC cell lines (H157, H2122, A549, H661 and H1299) and G_α16_ expression was quantified using the sense: caccacgctagcctggtcatg and anti-sense: gcgcccttcttgctgccctcggg primers. β-actin was used as an internal control for normalization. Data represents mean ± SEM of three separate experiments performed in duplicates. ^##^, *p*<0.01; versus control (Beas2B). B, Western blot analysis of G_α16_ expression in non-transformed and NSCLC cell lines. Equal amounts of total cell lysates of a non-transformed cell line (Beas2B) or NSCLC cell lines (H157, H2122, A549, H661 and H1299) were separated on a SDS-PAGE gels, transferred onto nitrocellulose blots and the “blots” were later probed with either anti-G_α16_, anti-G_αo_ or anti-β-actin antibodies.

We have identified G_α16_ and G_αq_ as important mediators of PPARγ and E-cadherin expression in NSCLC cell lines (Fig. 1). It was interesting to note that both G_α16_ and G_αq_ belong to the Gq family of G-proteins. However, since G_αq_Q209L expression not only showed less potent affects on PPARγ activity and E-cadherin expression but also had poor and inconsistent effects on NSCLC proliferation and anchorage-independent cell growth (data not shown), we focused on evaluating the role of G_α16_, but not G_αq_. In order to establish a potential role for G_α16_ in NSCLC, we first probed the expression levels of G_α16_ in a panel of NSCLC cell lines using quantitative RT-PCR (qPCR, Fig. 2A). For these experiments, total RNA was extracted from non-transformed lung bronchial epithelial cells (Beas2B), lung adenocarcinoma (A549, H2122), squamous cell carcinoma (H157) and large cell carcinoma cell lines (H661 and H1299), reverse transcribed and the cDNAs were later used to measure the levels of G_α16_ expression (Fig. 2A). Interestingly, G_α16_ expression was severely attenuated in all the NSCLC cell lines tested in comparison to non-transformed bronchial epithelial cell line (Beas2B, Fig. 2A). We also determined the protein levels of G_α16_ in the non-transformed lung bronchial epithelial cells (Beas2B) and NSCLC cell lines by using an antibody specific to G_α16_ (Fig. 2B)_._ Western blotting of NSCLC cell lysates revealed a complete loss in expression of G_α16_ (Fig. 2B). Although there is no detectable mRNA expression, H157 cells displayed some G_α16_ protein expression. Only a speculation, the detectable expression of G_α16_ in H157 might be due to low protein turnover. On the contrary, NSCLC cell lines and Beas2B expressed similar levels of G_αo_ (Fig. 2B), the G-protein that is specific to β-catenin dependent signaling pathway [2], [24]. Since, loss of heterozygosity (LOH) plays an important role during the inactivation of tumor suppressor genes (TSG), we also searched for LOH (segmented genotype intensity) at GNA15/16 locus (G_α16_) on our lung cancer cell lines using the CONAN (Copy Number Analysis) tool (http://www.sanger.ac.uk/cgi-bin/genetics/CGP/conan/) from the Sanger Cancer Genome Project. We could detect LOH in H157, A549, H2122 and H661. In addition, we also searched for somatic copy number variations (CNV) at GNA15/16 locus in human lung cancers. For this purpose, the CNV data in 493 lung adenocarcinoma and 416 lung squamous cell carcinoma patients were downloaded from The Cancer Genome Atlas (TCGA) (http://cancergenome.nih.gov/) Project. Gene-level copy number estimates were later processed using GISTIC2 [25] and the TCGA FIREHOSE pipeline. Strikingly, the mutation status of GNA15/16 locus was 1.22% for homozygous deletions and 53.14% for single copy deletions in human lung adenocarcinoma patients and 0.96% for homozygous deletions and 41.34% for single copy deletions in human lung squamous carcinoma patients. These data provide further evidence for the loss of G_α16_ in lung cancers.

### Role of G_α16_ in NSCLC Cell Proliferation and Anchorage-independent Cell Growth

**Figure 3:**
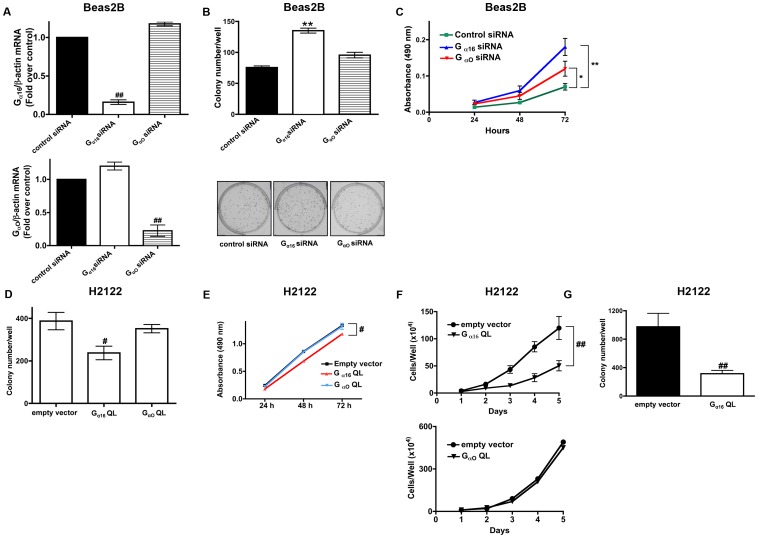
G_α16_ regulates NSCLC cell proliferation. A. Beas2B cells were transfected with either control siRNA or siRNAs specific to G_α16_ or G_αo_. Total RNA was isolated and analyzed for the expression of G_α16_ or G_αo_ using quantitative PCR. Normalized G_α16_ or G_αo_ mRNA levels to that of β-actin mRNA were represented in the graphs. ^##^, *p*<0.01; versus control siRNA. Beas2B cells were transfected with either control siRNA or siRNAs-specific to G_α16_ or G_αo_ and cell proliferation rates were later determined either by using a clonogenic assay (B) or an MTS assay (C) as described in the Methods. Upper panel represents mean ± SEM from two independent highly reproducible experiments, while representative images were displayed in the lower panel. Data represents mean ± SEM from three independent highly reproducible experiments. *, *p*<0.05; **, *p*<0.01; versus control siRNA. H2122 cells were transfected either with empty vector or constitutively active G_α16_Q212L or G_αo_Q205L expression vectors and the cell proliferation rates were later determined using either a clonogenic assay (D), an MTS assay (E) or five-day cell growth curve analysis (F) as described in the Methods. Upper panel represents mean ± SEM from two independent highly reproducible experiments, while representative images were displayed in the lower panel. Data represents mean ± SEM from three independent highly reproducible experiments. ^#^, *p*<0.05; G, H2122 cells were transfected with either empty vector or constitutively active G_α16_ Q212L and the abilities of the transfected cells to grow on soft agar were later probed. Data represents mean ± SEM from three independent highly reproducible experiments. ^##^, *p*<0.01; versus empty vector control.

Previous studies have established an important role for PPARγ in NSCLC cell proliferation, transformed growth, metastasis, and epithelial differentiation [23], [26]. Similarly, we also established the importance of Wnt7a/Fzd9 signaling in reduced transformed growth and increased cellular differentiation in NSCLC via the induction of PPARγ [5], [20]. If G_α16_ is an important mediator of Wnt7a/Fzd9 signaling, we reason that G_α16_ might also potentially mediate the transformed cell growth in NSCLC. In order to interrogate the specific involvement of G_α16_ in NSCLC cell proliferation and transformed cell growth, we utilized two approaches: (1) the effects of small interference RNAs (siRNAs) specific to G_α16_ and (2) expression of a constitutively active G_α16_Q212L. Small interference RNAs (siRNAs) specifically targeting either G_α16_ or G_αo_ were designed, tested for their specificity, and then employed to selectively suppress G_α16_ or G_αo_ in Beas2B cells. The siRNA reagents specifically suppressed either G_α16_ or G_αo_, achieving a 70% or more reduction in the expression of each protein (Fig. 3A). Scrambled siRNAs designed by the commercial supplier were tested as controls in some subsets; they showed no capacity to suppress either G_α16_ or G_αo_ expression (Fig. 3A). Treatment of non-transformed bronchial epithelial cells (Beas2B, with G_α16_ expression) with G_α16_-specific siRNAs significantly increased the cell proliferation as determined by clonogenic (Fig. 3B) and MTS cell proliferation assays (Fig. 3C). While, treatment of Beas2B cells with G_αo_-specific siRNAs, a G-protein that is specific for β-catenin-dependent signaling pathway [2], [24], had no or modest effect on cell proliferation rates, in comparison to control siRNA treated cells, as determined by clonogenic (Fig. 3B) and MTS cell proliferation assays (Fig. 3C), indicating a specific association of G_α16_ and NSCLC cell proliferation. If our hypothesis that G_α16_ mediates NSCLC cell proliferation were true, then expression of a constitutively active, GTPase deficient (Q212L) mutant of G_α16_ in NSCLC cells should result in reduced cell proliferation, even in the absence of Wnt7a. Indeed, transient expression of G_α16_Q212L, but not G_αo_Q205L, in H2122 cells resulted in reduced cell proliferation as determined by clonogenic (Fig. 3D), MTS cell proliferation assays (Fig. 3E), and/or 5-day cell growth curve analysis (Fig. 3F). In addition, stable expression of G_α16_Q212L in H2122 cells also inhibited the abilities of H2122 cells to grow on soft agar, an *in vitro* measure of cellular transformation (Fig. 3G). Thus, by using several distinct and powerful assays, we show that G_α16_ but not G_αo_ regulates NSCLC cell proliferation and transformed cell growth.

### Wnt7a-stimulated ERK5 Activation is G_α16_ Dependent

**Figure 4:**
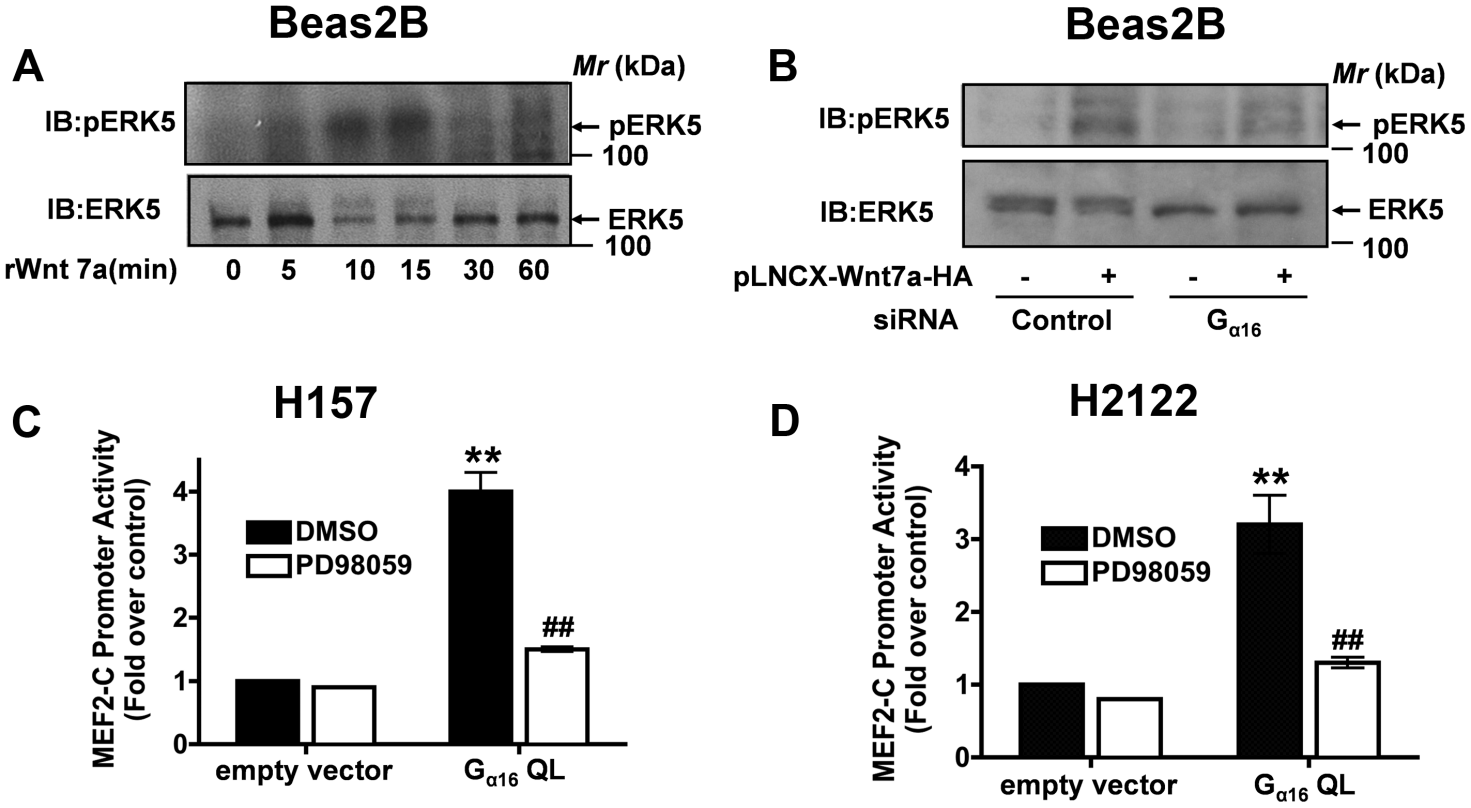
G_α16_ regulates Wnt7a/Fzd9-mediated ERK5 activation. A, Beas2B cells were serum starved for 2-PAGE gels and later probed for ERK5 activation by probing the nitrocellulose blots with anti-pERK5 antibodies and normalized for equal loading by probing with anti-ERK5 antibodies. B, Beas2B cells were transfected either with control siRNA or G_α16_-specific siRNAs together with or without Wnt7a expression vector. After 48 h, the cells were lysed and analyzed for ERK5 activation by probing the blots with anti-pERK5 and ERK5 antibodies. NSCLC cell lines, H157 (C) or H2122 (D) cells were transfected either with empty vector or G_α16_Q212L together with MEF2-C-dependent luciferase reporter, followed by a treatment either without or with MEK inhibitor PD98059 (20 µM). After 24 h, the lysates were assayed for luciferase activities as described in the Methods. Data represents mean ± SEM of three separate experiments. **, *p*<0.01; versus empty vector control. ^##^, *p*<0.01; versus G_α16_ Q212L.

We previously observed that expression of Wnt7a/Fzd9 in NSCLC cells results in robust activation of ERK5 [5]. We next tested if recombinant hWnt7a stimulation of Beas2B cells is also capable of activating ERK5; by immunoblotting SDS-PAGE gel blots with antibodies specific for phospho-ERK5 (Fig. 4A). Stimulation of Beas2B cultures with rWnt7a resulted in a rapid activation of ERK5 reaching a peak within 15 min of treatment (Fig. 4A). To ascertain if Wnt7a/ERK5 signaling was operating via G_α16_, we made use of G_α16_-specific siRNAs (Fig. 4B). In these studies, Beas2B cells were co-transfected with G_α16_-specific siRNAs either with or without Wnt7a expression vectors (Fig. 4B). Interestingly, treatment with G_α16_-specific siRNAs abolished the ability of Wnt7a to stimulate ERK5 activation (Fig. 4B). If depletion of G_α16_ could block Wnt7a-mediated activation of ERK5, then expression of constitutively active form of G_α16_ (Q212L) should induce ERK5 activation. In order to test this hypothesis, we made use of a MEF2-C-dependent luciferase reporter construct [27]. This reporter measures the ERK5 kinase activity, as it is obligate for activating MEF2-C-dependent gene transcription [27]. Transient expression of constitutively active form of G_α16_Q212L along with MEF2-C-luciferase reporter plasmids resulted in a robust increase in luciferase activities as determined in both H157 (Fig. 4C) and H2122 (Fig. 4D) NSCLC cell lines. Furthermore, the G_α16_Q212L-induced MEF2-C-dependent luciferase activities were sensitive to the treatment of PD98059, which inhibits MEK 1, 2, and 5 (Fig. 4C, D), but not by the MEK1/2 inhibitor U0126 (data not shown), indicating the specificity of our assay. Taken together, these data clearly establish that G_α16_ function is critical for Wnt7a-mediated ERK5 activation.

### G_α16_ Regulates Wnt7a-stimulated PPARγ Activation

**Figure 5:**
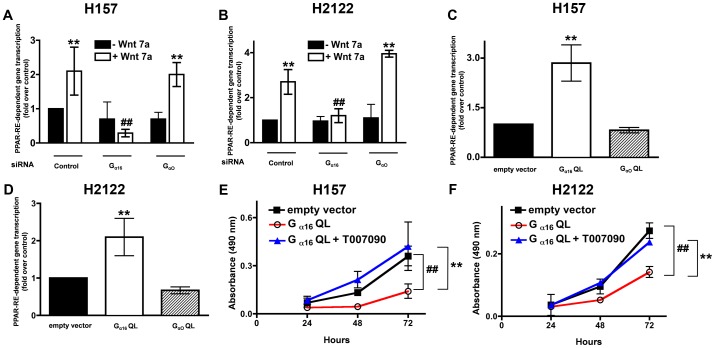
G_α16_ regulates Wnt7a/Fzd9-mediated PPARγ activation. NSCLC cell lines, H157 (A) or H2122 (B) cells were transfected either with control siRNA or G_α16_-specific siRNAs together with PPAR-RE-luciferase reporter and either without or with Wnt7a expression vector. After 48 h, the lysates were assayed for luciferase activities as described in the Methods. Data represents mean ± SEM of three separate experiments. **, *p*<0.01; versus empty vector control. ^##^, *p*<0.01; versus Wnt7a. NSCLC cell lines, H157 (C) or H2122 (D) cells were transfected either with empty vector or constitutively active G_α16_ Q212L or G_αo_ Q205L expression vectors together with PPAR-RE-luciferase reporter. After 48 h, the lysates were assayed for luciferase activities as described in the Methods. Data represents mean ± SEM of three separate experiments. **, *p*<0.01; versus empty vector control. NSCLC cell lines, H157 (E) or H2122 (F) were transfected with either empty vector or constitutively active G_α16_Q212L. After 24 h, the cells were treated either with or without PPARγ inhibitor (T0070907, 10 µM) as described in the Methods. Cell proliferation rates were later determined using an MTS assay as described in the Methods. Data represents mean ± SEM from three independent highly reproducible experiments. ^##^, *p*<0.01; versus empty vector control. **, *p*<0.01; versus G_α16_ Q212L+T007090.

We next interrogated if depletion of G_α16_ also blocks the far downstream signaling effector of Wnt7a/Fzd9 signaling *viz.,* PPARγ [5]. H157 and H2122 cells were co-transfected with G_α16_-specific siRNAs either with or without Wnt7a expression vectors and PPAR-RE luciferase reporter vector (Fig. 5A, B). Depletion of G_α16_, but not G_αo_, selectively blocked Wnt7a-stimulated PPARγ activation in both the cell lines tested (Fig. 5A, B). Consistent to the effects of G_α16_ depletion on Wnt7a-stimulated PPARγ activation, expression of constitutively active G_α16_Q212L, but not G_αo_Q205L, in H157 or H2122 cell lines induced a robust increase in PPARγ activity (Fig. 5C, D). Furthermore, G_α16_Q212L-induced anti-proliferative effects on H2122 cell growth were abrogated by intoxication of the transfected cells with PPARγ inhibitor (T007090) in both H157 (Fig. 5E) and H2122 cell lines (Fig. 5F). These data strongly suggest that the anti-proliferative effects of G_α16_ in NSCLC are mediated via ERK5 (Fig. 4) and PPARγ (Fig. 5).

### Novel Role for ROR1/2 in Wnt7a/Fzd9 Signaling

**Figure 6:**
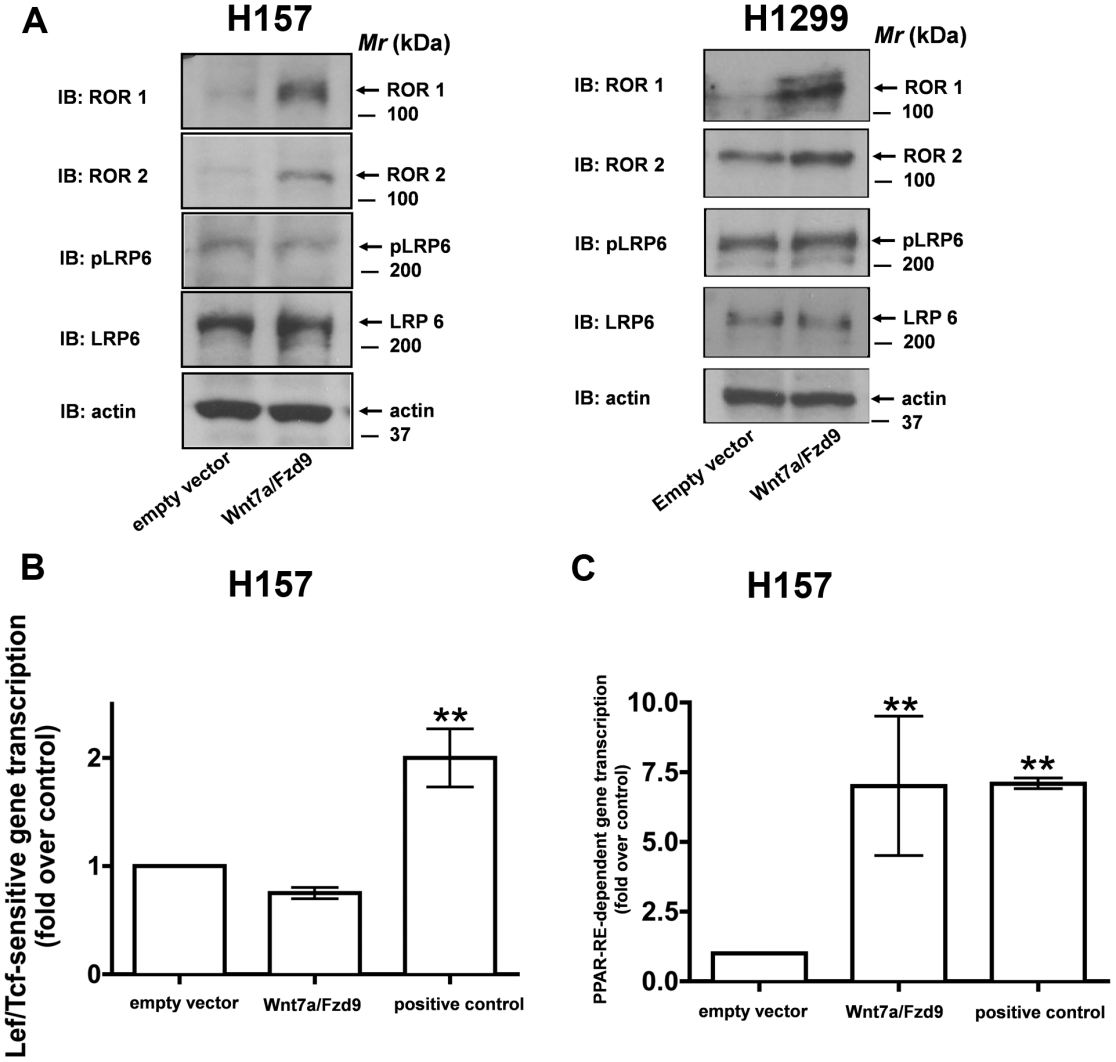
Wnt7a/Fzd9 signaling regulates ROR1/2 expression. A, H157 and H1299 cells were either transfected with empty vector or with Wnt7a and Fzd9 expression vectors. After 48-β-actin antibodies. H157 cells were transfected with either empty vector or Wnt7a and Fzd9 expression vectors along with either M50-TOPFLASH luciferase reporter (B) or PPAR-RE-luciferase reporter (C). Positive controls used in M50-TOPFLASH reporter experiments is the β-catenin expression vector and in the case of PPAR-RE-luciferase vector is the PPARγ expression vector. After 48 h, the lysates were assayed for luciferase activities as described in the Methods. Data represents mean ± SEM of three separate experiments. **, *p*<0.01; versus empty vector control.

It is well established that activation of Wnt/β-catenin-dependent signaling requires co-receptors low-density lipoproteins (LRP5/6) and the G-protein, G_αo_ [24], [28]–[30]. Since, we have established G_α16_ as a critical mediator of Wnt7a/Fzd9 signaling (Fig. 1, 2, 3,4), we next evaluated for the role of co-receptors, if at all, in mediating Wnt7a/Fzd9 signaling. For these studies, H157 and H1299 cells were transfected with either empty vector or Wnt7a and Fzd9 expression plasmids. Interestingly, probing the cell lysates expressing Wnt7a/Fzd9 revealed a robust increase in the expression of tyrosine-protein kinase orphan receptors, ROR1/2 (Fig. 6A). While, the co-receptors cardinal to Wnt/β-catenin-dependent signaling pathway, LRP6 or its activated form (phospho-LRP6-S1490) is unaffected (Fig. 6A). In strong support of our findings, Wnt7a/Fzd9 signaling also failed to stimulate TOPFLASH activity, a Wnt/β-catenin-specific read-out (Fig. 6B). While, under similar conditions, Wnt7a/Fzd9 stimulated a robust increase in PPAR-RE-dependent gene transcription, as expected (Fig. 6C). In total, these data suggest that the Wnt7a/Fzd9 signaling pathway, unlike that of β-catenin-dependent signaling mechanism, might signal via the G-protein, G_α16_ and the co-receptors, ROR1/2.

## Discussion

Wnt7a has been previously shown to be essential for the normal epithelium formation and for maintaining a normal epithelial phenotype in the lung [31]. Moreover, Wnt7a expression is frequently lost in NSCLC [32]. We have previously shown that re-expression of Wnt7a reversed cellular transformation, decreased anchorage-independent growth, and induced epithelial differentiation in NSCLC cells through its cognate receptor Fzd9 [5], [20]. This effect is mediated, at least in part, through ERK5-dependent activation of PPARγ [5]. Importantly, Wnt7a/Fzd9 does not activate the canonical Wnt/β-catenin signaling pathway. Frizzleds, members of the GPCR superfamily [33], display many of the landmarks observed in virtually all GPCRs, including the presence of seven hydrophobic transmembrane segments predicted to form alpha-helixes, and three intracellular loops as well as a cytoplasmic tail [33], [34]. Of note, Fzds are also reported as being closely associated with the adaptor molecules, like that of β-arrestins, a well-known adaptor protein involved in the GPCR de-sensitization [35] and regulators of G-protein signaling (RGS, [36]).

G-proteins are cardinal to GPCR signaling and have been shown to be involved in canonical Wnt/β-catenin signaling, non-canonical Wnt/Ca^2+^/cGMP pathway and planar cell polarity pathways [34]. Thus far, G_αo_ and G_αq_ were shown to be critical during mammalian development, and teratocarcinoma stem cell differentiation in response to oncogenic Fzd1 stimulation [24]. However, the tumor protective roles for G-proteins, if any, have not been identified. In the present study, we have identified G_αq_ family of G-proteins as novel downstream mediators of Wnt7a/Fzd9-mediated activation of ERK5 and the tumor suppressor gene PPARγ. For the first time, we show that G_αq_ family members, specifically, G_α16_, can activate a novel non-canonical Wnt signaling. The signaling cascade downstream of G_α_ proteins involves diverse and complex kinases that ultimately lead to regulated gene transcription and changes in cell physiology. Each G_αq_ family member has been implicated in regulating one or more of the mitogen-activated protein kinase (MAPK) pathways in cell cultures, although the precise mechanism/s of signaling remains unclear. In the present study, we also show that G_α16_ is the downstream effector of Wnt7a/Fzd9 signaling that leads to the activation of ERK5. Interestingly, other studies have shown that GPCRs also can stimulate ERK5 through mechanisms that involve G_αq_ and G_α12/13_, independently of Rho, Rac1 and Cdc42 [37]. However, we did not see any activation of PPARγ by G_α12_ expression (Fig. 1). Thus far, G_αq_-mediated MAPK signaling is restricted to the activation of JNK1/2 or ERK1/2, but not ERK5. In the present study, we also show that a member of G_αq_ family, G_α16_, as a novel regulator of ERK5. It is well known that the G_αq_ family (G_αq_, G_α11_, G_α14_, G_α15/16_), upon activation, binds and stimulates PLC-β-mediated inositol phosphate signaling cascade, which leads to calcium mobilization and PKCα activation via phospholipid phosphatidylinositol bisphosphonate (PIP2), inositol triphosphate (IP3) and diacyl glycerol (DAG, [38]). In the same lines, Wnt7a/Fzd9 signaling also stimulated PLCβ and PKC (data not shown). Interestingly, PKCα has been shown to be associated with reduced cancer cell growth via inhibition of S-phase and up regulation of p21 [39]. Moreover, interaction between PKC and ERK5 have been suggested by Li *et al.,* demonstrating that typical PKC regulate cytokines through MEKK2/ERK5-dependent and independent pathways [40]. Furthermore, atypical PKCs have also been shown to activate MEK5/ERK5 [40], [41]. However, in the current study we did not investigate the association between PKC and ERK5. Thus, further evaluations are necessary to reveal the precise mechanism/s of anti-tumorigenic effect of the G_αq_ family in NSCLC cells.

In the present study we identify G_α16_ as a novel regulator of NSCLC cell proliferation and anchorage-independent cell growth. We show that the expression of G_α16_ is lost not only in NSCLC cell lines but also in human lung adenocarcinoma and squamous carcinoma patients (Fig. 2). Likewise, Wnt7a expression is also lost in lung cancers [6], [32], [42]. However, it is not known if a synergy exists between loss of G_α16_ and other tumor suppressor genes like Wnt7a, P53, and PTEN, and therefore awaits further study. It was shown earlier that G_α16_ signaling leads to reduced cell growth in small cell lung cancer (SCLC), which accounts to only 20–25% of primary lung cancers [11]. Similarly, in contrast to NSCLC, growth of small cell lung cancer cells (SCLC) is driven through the establishment of neuropeptide autocrine loops [43]. Numerous studies have shown that the mitogenic signal driven by this autocrine loop is mediated through G_αq_ [43]. Thus, activation of G_αq_ plays opposing roles in distinct types of lung cancer, a pro-tumorigenic role in SCLC and potentially an anti-tumorigenic role in NSCLC, via activation of non-canonical Wnt signaling. Thus, use of drugs targeting GPCRs or G_αq_-protein family might represent a novel therapeutic strategy in treating specific subtypes of lung cancer.

It is well known that PPARγ receptors are expressed in a variety of tumor cells and activation of PPARγ with ligands leads to either inhibition of cell proliferation or by induction of apoptosis [44]. Furthermore, thiazolidinediones (TZDs), a class of anti-diabetic drugs and synthetic ligands for PPARγ, were also utilized in anti-cancer therapies [45], [46]. It was shown that TZDs not only reduced the proliferation rates of A549 cells in *in vitro* cell cultures but also reduced A549-induced tumors in nude mice [47]. In the present study, we also identify G_α16_ as a novel regulator of PPARγ (Fig. 1). Therefore, therapeutic interventions to restore the lost functions of G_α16_ in lung cancers can compliment the TZD-based anti-cancer therapies.

**Figure 7:**
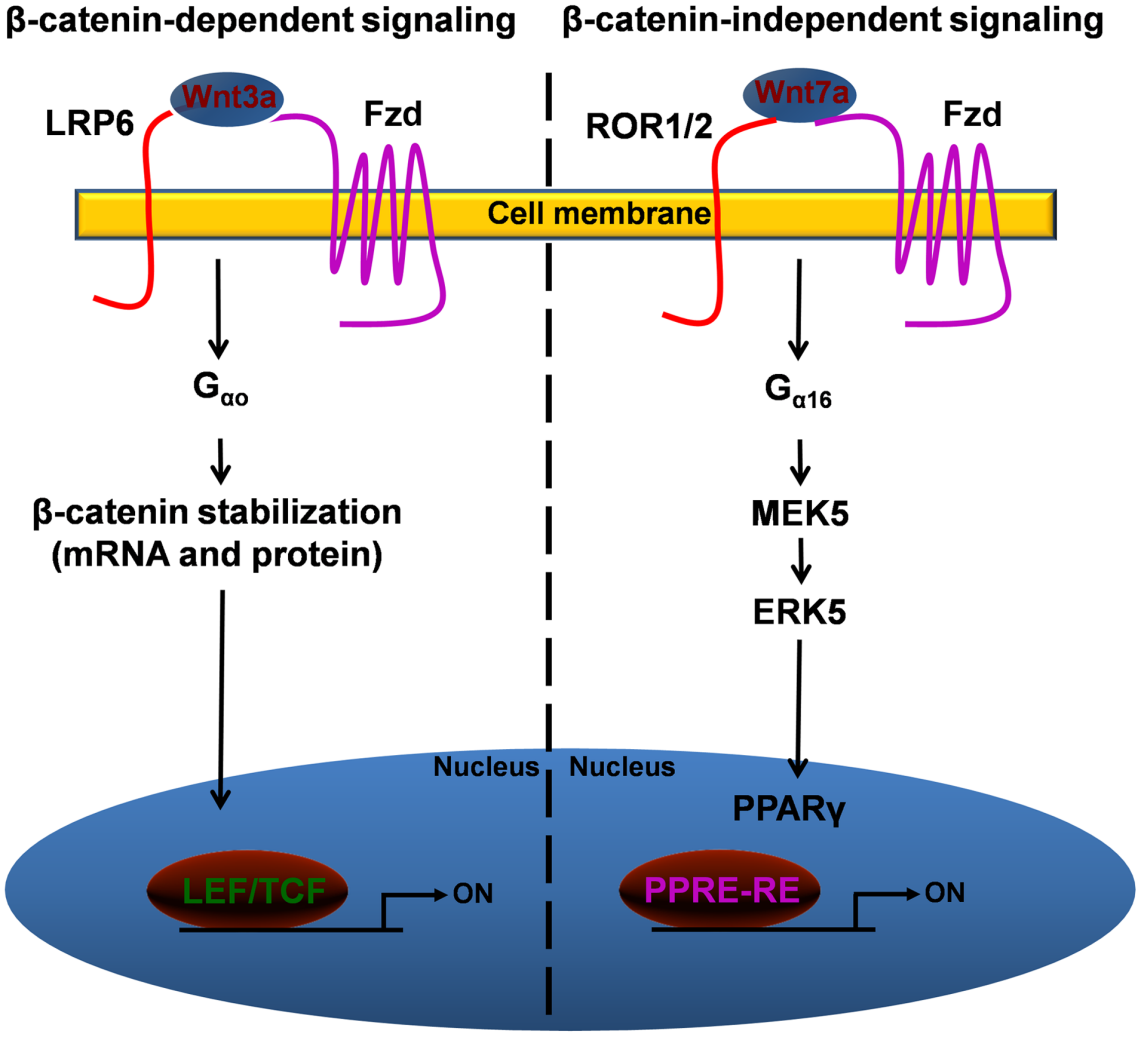
Schematic representation of differential utilization of co-receptors and G-proteins for β-catenin-dependent and β-catenin-independent signaling pathways. Based on our current understanding of Wnt-sensitive pathways, we propose that Wnt binding to Fzd and co-receptor LRP5/6, and mediated by G_αo_, leads to activation of β-catenin-dependent gene transcription. While, Wnt binding to Fzd and co-receptor ROR1/2, and mediated by G_α16_, leads to the activation of β-catenin-independent signaling pathway, mainly via activation of ERK5 and PPARγ.

The current study also reveals novel roles for upstream effectors like that of ROR1/2 in Wnt7a/Fzd9 signaling. Our results also hint at how cell signaling networks might be differentially utilizing various co-receptors and/or G-proteins in regulating β-catenin-dependent and β-catenin-independent signaling networks (Fig. 7). In summary, our data reveal a novel connection between Wnt7a/Fzd9 signaling and the G-protein G_α16_ and co-receptors ROR1/2 in mediating the stimulation of ERK5-dependent activation of the tumor suppressor gene PPARγ. ERK5 regulates a growing number of nuclear transcription factors that maybe associated with tumor growth suppression, thus identification of novel G-protein-mediated activation of ERK5, like that of G_α16_, could be an attractive therapeutic target. Thus, our emerging knowledge of GPCRs and intensive current drug development pipelines, place this family of receptors firmly in the center of attention as potential candidates for future cancer therapies.

## Materials and Methods

### Cell Culture and Inhibitors

A human non-transformed lung epithelial cell line (Beas2B) and NSCLC cell lines (H2122, H157 and H1299) were obtained from ATCC (Manassas, VA, USA). All the cell lines were cultured in RPMI 1640 medium (10-040-CV, Cellgro, Mediatech Inc., Manassas, VA) supplemented with 10% fetal bovine serum (FBS) in a humidified 5% CO_2_ incubator at 37°C. Stable transfectants of H2122 (H2122-LNCX and H2122-G_α16_Q212L) were made using retroviral mediated gene transfer as previously described (Winn et al., 2005; Wick et al., 2002). The cell lines were cultured bi-weekly and stocks of cell lines were passaged no more than ten times for use in experiments. The inhibitors used in our studies include, MEK inhibitors, [PD98059 (Sigma), U0126 (CalBiochem)] and PPARγ antagonist (T0070907, Calbiochem/EMD Biosciences).

### Cell Proliferation Studies and Anchorage-independent Growth

Clonogenic assays were performed in triplicates by seeding 1000 cells in a well of 12-well culture plate followed by incubation at 37°C in a 5% CO_2_ incubator. After 72 h, cell colonies were stained using a staining solution (0.5% of Crystal Violet, 12% Gluteraldehyde, 87.5% of water) for 1 h at room temperature. After de-staining in water and drying, colonies were quantified using Biorad Chemidoc Imaging System. Cloning efficiency represents the mean number of colonies formed per well.

MTS assays were performed in duplicates by seeding 500 cells in a well of 96-well culture plate, followed by incubation at 37°C in a 5% CO_2_ incubator. Cell proliferation was measured after 24, 48 and 72 h by adding 20 µl of MTS reagent (Cell Titer 96® Aqueous One Solution, G3582, Promega Corporation, Madison, WI) to each well, followed by incubation at 37°C. After 1 h, the absorbance of the formazon product was measured at 490 nm using a plate reader. Normalized absorbance values (sample readings-readings of media only blank) were represented in the graphs.

For measurement of cell growth rates, 50,000 cells in complete growth medium were seeded per well in a 24-well culture plate. On subsequent days, cells were trypsinized from the wells with 100 µL of trypsin, diluted with 400 µL of growth medium, and counted using a hemocytometer.

For measurement of anchorage-independent cell growth, 5,000 cells were plated in triplicates in 35-mm wells of a six-well plate in a volume of 1.5 ml of growth medium containing 0.3% noble agar onto a base of 1.5 ml of growth medium containing 0.5% agar. The plates were incubated in a 37°C CO_2_ incubator for 14 days. Later, colonies were stained for 5–16 h at 37°C with nitroblue tetrazolium chloride (1 mg/ml), visualized under a microscope, and counted.

### Transfections and Luciferase Reporter Assays

The reporter plasmids (PPAR-RE-luciferase reporter, E-cadherin promoter-luciferase-reporter, or MEF2-C-promoter-luciferase reporter, expression plasmids (pLNCX-Wnt7a-HA and pLPCX-Fzd9) and CMV-β-galactosidase control plasmids were transiently transfected into NSCLC cells using LipofectAmine reagent (18324-012, Invitrogen, Carlsbad, CA, USA) as per the manufacturer’s recommendations. The MEF2-C-promoter luciferase reporter was a kind gift from Dr. Rebecca Schweppe (University of Colorado), pGL2 basic-Ecad K1 E/E/E-luciferase reporter was a kind gift from Dr. Eric Fearon (University of Michigan), PPAR-RE-Luciferase was a kind gift from Dr. Raphael Nemenoff (University of Colorado), and pMV-7 plasmids encoding constitutively active forms of G-proteins were a kind gift from Dr. Lynn Heasley (University of Colorado). All of the luciferase activities were normalized to CMV-β gal activities. The expression plasmid for pLNCX-Wnt7a-HA was a gift from Dr. Jan Kitajewski (Columbia University) and pLNCX-Wnt3 was a gift from Dr. Randall Moon (University of Washington). For studies involving the use of MEK inhibitors PD98059 (20 µM, Sigma) or U0126, (10 µM, Calbiochem/EMD Biosciences, San Diego, CA), H2122 and H157 cells were co-transfected either without or with G_α16_Q212L and MEF2-C-luciferase reporter plasmids followed by treatment with MEK inhibitors. After 24 h, the lysates were assayed for luciferase activities.

### G-protein Knock down Studies

Beas2B cells or NSCLC cells (H157 or H2122) were seeded in a 100 mm dish (2×10^6^ cells), followed by incubation for 1 h at 37°C. G-protein specific siRNAs (5 nM) diluted in 1 mL of serum free medium were mixed with 40 uL of Hiperfect Transfection Reagent. After incubation of the siRNA complexes for 5 min at room temperature, the siRNA complexes are added drop-wise onto the cells. The cells were incubated for 48 h and analyzed for G-protein knock down. While, the control siRNAs (Qiagen, all stars negative control, #1027280) and G_αo_ specific siRNAs (Qiagen, # SI00128632) were pre-designed, the G_α16_-specific siRNAs were custom synthesized from Qiagen. The sequences of the G_α16_-specific siRNAs are as follows: sense strand-GGUUCAUCCUGGACAUGUATT and anti-sense strand-UACAUGUCCAGGAUGAACCTC.

### Immunoblot Analysis

Cell extracts were prepared in a lysis buffer (0.5% Triton X-100, 50 mM β-glycerophosphate, pH 7.20, 0.1 mM sodium vanadate, 2 mM MgCl2, 1 mM EGTA, 1 mM dithiothreitol, 2 µg/ml leupeptin, and 4 µg/ml aprotinin) and the western blot analysis was carried out as previously described [26]. The following antibodies were used for immunoblotting: G_αq_, G_αo_, G_α16_ (Santa Cruz), phospho-ERK5 and total ERK5 (Cell Signaling). Aliquots of various NSCLC extracts were resolved on 10% SDS-PAGE gels and transferred to nitrocellulose. The filters were blocked in Tris-buffered saline (10 mMTris-Cl, pH 7.4, 140 mMNaCl, containing 0.1% Tween 20 (TTBS) and 3% nonfat dry milk and then incubated with the same blocking solution containing the indicated antibodies at 0.5 µg/ml for 12–16 h. For immunoblotting of phospho-ERK-5, filters were blocked in TTBS containing 3% bovine serum albumin. The filters were extensively washed in TTBS, and bound antibodies were visualized with horseradish peroxidase (HRP)-coupled secondary antibodies.

### Data Analysis

Data were compiled from at least three independent, replicate experiments, each performed on separate cultures and on separate occasions. The responses are displayed as “fold-changes”. Comparisons of data among experimental groups were performed using student’s *t*-test for assessing variance. Increase in statistical significance (*p* value of <0.05) is denoted with an “*” symbol, while a decrease in statistical significance (*p* value of <0.05) is denoted with a “#” symbol.
